# Department of Surgery

**Published:** 1898-12

**Authors:** L. A. Merillat, W. E. A. Wyman

**Affiliations:** Secretary of M’killip Veterinary College, Chicago


### DEPARTMENT OF SURGERY.

By L. A. Merillat, V.S.,
SECRETARY OF M'KILLIP VETERINARY COLLEGE, CHICAGO.

WITH THE COLLABORATION OF

W. E. A. Wyman, M.D.V., V.S.,
MILWAUKEE, WIS.,

AND OTHERS.

### Anæsthetics.

*General anæsthetics.* Chloroform for the horse and ox, and ether for the dog and cat.

The administration of general anæsthetics is of special importance to the surgeon who undertakes capital operations and seeks the best results therefrom. Minor operations, however painful, are readily performed under topical anæsthesia, or even without its assistance; but prolonged, tedious, complicated, or delicate ones can, on the whole, be performed successfully only under the influence of general anæsthesia. The advantages of general anæsthetics in such operations are summarized as follows:

1. The field of operation can be more thoroughly and easily disinfected.
2. The incisions can be more accurately made.
3. Hemorrhage is more readily controlled.
4. Sutures, bandages—in fact, all dressings—are applied to better advantage.
5. The patient suffers no pain.
6. The seat of operation can be placed in the most advantageous position.
7. The procedure is a better exemplification of the surgeon's skill.

The disadvantages are confined, first, to the loss of time required for administration, and, secondly, to the danger of death. The former may be dismissed by adding that surgical anæsthesia can be produced in the domestic animals in from twenty to thirty seconds, with both ether and chloroform. Then the subsequent steps of the operation, uninterrupted by struggling, are so facilitated that the ten to fifteen minutes required for the patient to revive and regain his feet are not actually lost.

As to the danger of death, records of approximately 15,000 cases show but four deaths, all of which were traced, not to cardiac lesions, as might be expected, but to careless administration, especially in failing to observe the arrested respiration in time to apply restorative measures.

*Physiological Action of Chloroform.* The action of chloroform is central. The phenomena following its rapid introduction into the system through the aerial mucous membrane is doubtless due to a *transient* alteration of the nerve-cell protoplasm of the *cerebrum* and subsequently the *spinal centres.* The theory that it retards the elimination of $CO_2$ is no longer accepted. The horse and ox respond more readily and more effectually to chloroform inhalation than man because the area of absorbing-surface furnished by the aerial membrane is proportionately larger in the former. Chloroform is so volatile, has so large a surface through which to enter the circulation rapidly, and finally so small a structure to act upon, that its action in the horse and ox is almost instantaneous.

Its action on the nerve-centres immediately gives rise to the following generalized manifestations named in the order of their occurrence:

1. Relaxation of the voluntary muscles and blunted consciousness.
2. Total loss of consciousness.
3. Loss of sensibility and total loss of voluntary motion.
4. Loss of involuntary motion—death.

The very first effect of chloroform inhalation is violent struggling induced by the sense of suffocation, irritation of the upper air-passages, and the effort to resist a deep inspiration. This has been described as the *stimulating stage*—a name it scarcely deserves.

The study of anæsthesia in the domestic animal should begin with the first deep inspiration, because immediately after the first deep inspiration anæsthesia proper commences, and is readily recognized by a partial relaxation of the entire muscular system. The second and third inspirations place the voluntary muscles completely at rest. At this stage—*the narcotic*—the functions are only blunted, consciousness in only partially lost, reflex activity is only barely impaired, and the observable involuntary functions (circulation and respiration) are, if anything, exalted. At this period the animal is permitted to freely inhale two or three inspirations of pure air; the symptoms already induced will almost immediately disappear. This phenomenon beautifully illustrates the *rapid* as well as the *transient* action of chloroform. On the other hand, two to four chloroform inhalations added to those already given promptly bring the patient into complete unconsciousness, and total loss of reflex activity. The involuntary functions are only diminished. This is the condition to be sought in performing operations, and is designated the *surgical stage.*

The number of inspirations required to arrest the involuntary functions after the surgical stage varies considerably. Ten inspirations are often sufficient to arrest respiration while in some instances the patient succumbs only after twenty-five to thirty. The toxic effects are respiratory, rapidly followed by cardiac arrest. In no case in the horse are these effects reversed. Chloroform killed by respiratory arrest in all cases experimentally destroyed.

A very essential point to be remembered in chloroformization is that the animal most susceptible is the least liable to succumb, while the one difficult to anæsthetize must be treated guardedly. The horse is only in rare cases difficult to anæsthetize, probably on account of the normal condition of the nervous system as compared with the "alcoholic" condition so common in the homo. It is a well-known fact that the drunkard can be anæsthetized only with the greatest danger, and in many case true surgical anæsthesia cannot be produced at all, but, instead, violent struggling ensues during the whole period of administration. Such poor results are never seen in the horse or ox, but, on the contrary, they all yield to its influence promptly and with a minimum degree of danger.

*Administration.* Chloroform should, if possible, be administered by an experienced assistant, who must be instructed to take no part or interest in the operation. He must adopt a method of administering large quantities of chloroform at each inspiration, and guard only over the patient's respirations and cornea. Too much stress must not even be placed upon the latter, because a patient may respond to a touch on the cornea when the respirations are already too low to stand another inspiration of chloroform. The cornea shows the condition of anæsthesia rather than the state of the involuntary functions. In other words, the cornea must be regarded as the proper and only guide as to whether or not the patient is ready for the surgeon's knife, while the respiration furnishes the protection to the patient's life. The surgeon may often command: "Administer more chloroform; the patient is resisting;" and the assistant will answer: "Doctor, I cannot; the respirations are faulty." This is a very common occurrence, and in such a case the operation must proceed without perfect anæsthesia. Under no circumstances must the assistant be influenced by such requests, because the surgeon has usually been too much occupied with his work to have noted the patient's condition.

The veterinarian who has no assistant is under some disadvantages unless the operation can be completed in a few moments. In the latter instance the animal is perfectly anæsthetized and the operation completed before the patient offers much resistance. In the case of prolonged procedures the veterinarian who is so situated should call on his neighbor veterinarian for assistance. The surgeon who assists in casting and preparing a patient without experienced help, and must then administer an anæsthetic through a prolonged operation, is in no condition to perform the duties of a surgeon.

*Mode of Administration.* The animal is cast and securely tied in the costal position. A rubber sheet, one metre square, is placed under the head. A coarse sponge, ten to fifteen centimetres in diameter, is sprinkled with ninety cubic centimetres of chloroform. The administrator lies upon the patient's head, gathers the sheet over the nose, holds the under nostril shut with one hand and the sponge over the upper nostril with the other. The hand closing the under nostril is outside the sheet, and is also engaged in holding the sheet tightly over the maxillary region to exclude all air. The hand holding the sponge is, of course, inside the sheet, and is held a short distance from the nostril. Immediately the animal will struggle violently and utter somewhat unpleasant groans, but the weight of the body upon the poll and the under hand raising the nose from the floor will modify the struggles of the head to a suitable degree. This position must be maintained until the patient is forced to take a deep inspiration, after which the muscles will relax and the struggles become less violent. Two or three subsequent inhalations will produce complete relaxation, but the administrator must continue "on the offensive" during three or four more inspirations. During the latter the animal sinks rapidly into unconsciousness and insensibility. The cornea no longer promptly responds to the touch, and the respirations have become soft and shallow; in fact, the patient is ready for the operation.

The bedding, which has been disturbed by the struggles, is replaced under the head and the rubber sheet spread out anew, while the surgeon is exposing and preparing the seat of operation. The administrator now assumes a more comfortable position by sitting upon the poll (when the operation is not in that region), from which position the respirations can be studied both at the abdominal muscles and nostrils. An occasional touch to the cornea shows the condition of anæsthesia. But I have always made it a point to withhold the chloroform as long as the patient offers no resistance to the operator, even when the cornea became sensitive. This I offer as a suggestion to the inexperienced, who are always more or less fearful of causing death. With the very first evidence of returning sensibility the sponge is again sprinkled with 15 or 20 c.c. and applied to the upper nostril, but the sheet is left spread out and air allowed to pass freely through the under nostril. If these simple methods are followed no deaths will ever occur.

Summary. Take no interest in the operation, but watch the patient's respirations.

2. Administer the chloroform rapidly and incessantly and without air, until the patient is completely anæsthetized; then administer only through one nostril, with air passing freely through the other.

3. Do not place too much stress upon the cornea.

4. Do not administer when the respirations are not deep and regular.

5. The heart will take care of itself.

Ether is quite as effectual in the dog and cat for general anæsthesia, but the time required to produce complete anæsthesia is somewhat longer than chloroform in the horse.

### *Topical Anæsthesia.*

Topical anæsthesia is produced in veterinary practice chiefly by the use of cocaine and eucaine. The latter has nothing to recommend it over the former, in the horse. Cocaine in a 10 per cent. solution can be used with safety in all forms of superficial operations, and if the surgeon is mindful of the maximum dose no bad result is possible. Cocaine paralyzes all sensory nerves with which it comes in contact; others can do no more.

L. A. MERILLAT.

---

CHARLES WEISS, of Somerville, Mass., has petitioned the Boston Board of Aldermen for a license to slaughter horses for the domestic and foreign meat-trade, "the product to be sold or used for food, packed in barrels for export." There is no law against the practice, but this is the first time it has come up for legislative consideration. A large export trade in horsemeat is carried on outside of Massachusetts, the product going mainly to Germany, though its use is increasing in all Continental countries.

